# Chronic inflammatory arthritis in 22q11.2 deletion (DiGeorge) syndrome: a multicentric study

**DOI:** 10.1186/s13023-025-04088-2

**Published:** 2025-11-24

**Authors:** Emily Liebling, Caroline Freychet, Valentina Guarnieri, Marija Jelusic, Jordi Antón López, Tilmann Kallinich, Davide Montin, Liza J. McCann, Brigitte Bader-Meunier, Terence Blaine Crowley, Irene Lemelle, Donna McDonald-McGinn, Mercedes Serrano, Jean Louis Stephan, Chiara Azzari, Gabriele Simonini, Teresa Giani

**Affiliations:** 1 Children’s Hospital of Philadelphia, Philadelphia, USA; 2 Department of Pediatric Rheumatology, Necker-Enfants Malades Hospital, AP-HP, Paris, France; 3 Reference Center for Rheumatic, AutoImmune and Systemic Diseases in Children (RAISE), Imagine Institute, Inserm U 1163, Paris, France; 4 Paediatric Rheumatology Unit, Meyer Children’s Hospital IRCCS, Florence, Italy; 5 Immunology Unit, Department of Pediatrics, Meyer Children’s Hospital IRCCS, Florence, Italy; 6 Pediatrics, University Hospital Centre Zagreb, Zagreb, Croatia; 7 Pediatric Rheumatology, Hospital Sant Joan de Déu, Universitat de Barcelona, Barcelona, Spain; 8 Pediatric Rheumatology, Charitè, Berlin, Germany; 9 Pediatrics and Infectious Diseases, Regina Margherita Children’s Hospital, Torino, Italy; 10 Pediatric Rheumatology Department, Alder Hey Children’s NHS Foundation Trust, Liverpool, UK; 11 Paediatric Onco-Haematology, University Hospital of Nancy-Brabois, Vandoeuvre-Lès-Nancy, France; 12 Pediatric Neurology, Hospital Sant Joan de Déu, Barcelona, Spain; 13 Pediatric Department, University Hospital Saint Etienne, St-Priest-en- Jarez, France; 14 Rheumatology Unit, Meyer Children’s Hospital IRCCS, Viale Pieraccini, 24, Florence, Italy

**Keywords:** 22q11DS, DiGeorge syndrome, Arthritis, Autoimmunity, Juvenile idiopathic arthritis

## Abstract

**Background:**

22q11 deletion syndrome (22q11DS) is a genetic disorder caused by a deletion at chromosome 22q11.2. Chronic arthritis may be occasionally observed in these, if this manifestion represents a concomitant association of two diseases or a rare complication of Di George syndrome is still a debate. This study aims to describe a retrospective, multicenter analysis of patients with 22q11DS, who developed chronic inflammatory arthritis, focusing on clinical presentation, diagnosis, and management.

**Methods:**

An e-mail survey was distributed to pediatric centers, identifying patients diagnoses with both 22q11DS and chronic arthritis from 1992 to 2024. The clinical course was documented through medical record review.

**Results:**

A total of 30 patients were identified with a female predominance (20 cases). The median age at 22q11DS diagnosis was 12 months, and 3 years at arthritis onset. Polyarticular involvement was observed in 50.0% of patients, and the median number of affected joints at onset among all patients was 4.5 (range 1–25). Only one child developed uveitis. Erythrocyte sedimentation rate was elevated in 72.0% of tested patients, and C-reactive protein in 83.3%. Antinuclear antibodies was negative in 23.3% of patients, while rheumatoid factor (RF) and cyclic citrullinated peptide (CCP) antibodies were consistently negative. Treatment included systemic glucocorticoids (53.3%), methotrexate (66.6%), and biologic agents (56.7%). Infections were generally mild, primarily affecting the respiratory tract. At last follow-up, arthritis persisted active in 53.3% of cases, with 78.6% of those in remission still on medication.

**Conclusions:**

Arthritis in 22q11DS appears to be a distinct clinical entity characterized by an aggressive phenotype and a severe, prolonged course, typically lacking CCP and RF antibodies and rarely associated with uveitis. These features suggest a distinct clinical entity, diverging from classical juvenile idiopathic arthritis.

## Introduction

22q11 deletion syndrome (22q11DS) is a genetic condition caused by a heterozygous microdeletion on chromosome 22q11.2 [1]. With an estimated incidence of 1 in 4.000 live births, it is the most prevalent human deletion syndrome, and in nearly 90% of cases, the genetic defect arises *de novo* [2, 3]. Although the deleted region is consistent, the phenotypic expression among affected individuals varies considerably [2]. The syndrome encompasses conditions such as DiGeorge syndrome, velo-cardio-facial syndrome, and conotruncal anomaly face syndrome, all of which share an overlapping region on chromosome 22q11 and exhibit similar phenotypes [4]. Reduced 22q11 gene dosage induces aberrant embryonal pharyngeal arch and pouch development, leading to a spectrum of congenital malformations, including cardiac defects, dysmorphic facies, skeletal abnormalities, and abnormal thymic development. Additionally, the deletion may also contribute to neurodevelopmental disorders [5].

Currently, molecular diagnosis of 22q11DS is mainly performed by chromosomal microarrays analysis. However, various techniques, such as fluorescence in situ hybridization (FISH), multiplex ligation-dependent probe amplification (MLPA), and restriction fragment analysis using Southern blots, are also available [6]. T-cell receptor excision circles (TREC) testing on dried blood spots has been recently introduced in some countries as part of a newborn screening programs, to enable a more timely diagnosis of inborn error of immunity (IEI) [7].

Up to 77% of patients show some degree of immune dysfunction. While severe immunodeficiency, resulting from complete thymic aplasia, occurs in less than 1% of patients, immune dysfunction is extremely frequent and does not necessarily correlate with the degree of thymic hypoplasia [8].

Thymic defects reduce T cell receptor (TCR) diversity and impair control of regulatory T (Treg) cells, resulting in impaired self-tolerance, increased expansion of peripheral naïve CD4 T cells and a distorted Treg memory/activation phenotype, which predisposes individuals to infections, atopy, malignancies and autoimmunity [9–11]. The mechanisms behind autoimmune manifestations in 22q11DS are complex and still largely uncharacterized, likely involving both cellular and humoral immune pathways [8, 12].

Various autoimmune conditions, including autoimmune thyroiditis, vitiligo, and arthritis, have been reported in 22q.11DS patients. Emerging evidence also suggests that psychiatric symptoms and cognitive impairment may have an autoimmune underpinning [13–18].

Inflammatory arthritis in children with 22q11DS was first described in 1996 [19]. Two girls with velopharyngeal insufficiency developed severe polyarticular arthritis at 7 and 12 years of age, despite the absence of a clear clinical or laboratory evidence of immunodeficiency. Since then, additional cases of arthritis in 22q11DS have been published, raising debate over whether arthritis is considered a coincidental association or a rare complication of this syndrome.

Herein we present a retrospective, multicenter analysis of patients with 22q11DS who developed chronic inflammatory arthritis. We report clinical presentation and management strategies of chronic arthritis in this population.

## Methods

A consortium of international pediatric centers developed an observational retrospective database of patients with 22q11DS and arthritis. The inclusion criteria were (1) the molecular diagnosis of 22q11DS; (2) chronic inflammatory arthritis in one or more joints, persistent more than 6 weeks and with a disease onset before 16 years of age. The exclusion criteria included (1) lack of molecular confirmation of 22q11DS; (2) arthritis not meeting the definition of chronic inflammatory arthritis or alternative diagnoses explaining joint symptoms; (3) Insufficient availability of data in the databases.

Patient data related to 22q11DS and chronic arthritis were retrospectively collected from medical records spanning 1992–2024 and entered into a dedicated dataset. Demographic, clinical and laboratory data related to the 22q11DS, and clinical and laboratory details concerning chronic arthritis were required in the survey questionnaire. Since anonymized data were used and the study was retrospective, ethics approval was obtained only where required by local regulations.

Descriptive statistics were performed, reporting means, medians, ranges, and absolute and relative frequencies for each variables.

## Results

This study recruited 30 patients affected by 22q11DS associated with chronic arthritis followed from 1992 to 2024 in 9 different pediatric centers: Children’s Hospital of Philadelphia, Philadelphia, USA; Necker Enfants Malades, Paris, France; Pediatric Rheumatology, Nancy, France; Pediatric Rheumatology Unit, Meyer Children Hospital IRCCS, Firenze, Italy; Pediatrics and Infectious Diseases, Regina Margherita Children’s Hospital, Torino, Italy; Pediatrics, University Hospital Centre Zagreb, Croatia; Pediatric Rheumatology Department, Alder Hey Children’s NHS Foundation Trust, Liverpool, United Kingdom; Pediatric Rheumatology, Hospital Sant Joan de Déu, Barcelona, Spain; Pediatric Rheumatology, Charitè, Berlin, Germany. Four of the included patients had been previously reported [16, 20].

All patients had a laboratory confirmed chromosomal aberration, observed with chromosomal microarray studies in 7 cases, FISH analysis in 21 cases, and MLPA analysis in 2 cases. Ten patients were male. The mean age at 22q11DS diagnosis was 27 months (median, 12 months; range, birth to 180 months); the majority of patients (18/30) were diagnosed within the first year of life. Twenty-one patients (70.0%) were female. A positive family history for 22q11DS was documented in 4 cases (father and brother in one case, mother in a second patient, father in a third patient, and mother and brother in a fourth one). All cases showed at least two out of the five major characteristics of 22q11DS (heart malformations, facial dysmorphism, T-cell immuno-deficiency, palatal clefts, and hypocalcemia/hypoparathyroidism). The most common major feature of 22q11DS was facial dysmorphism (96.6%), followed by congenital heart malformations (80.0%), hypocalcemia or hypoparathyroidism (43.3%), T-cell deficiency (33.3%), and palatal cleft (16.7%).

Among the 30 patients, beyond these major features, additional clinical manifestations were recorded. Developmental delay was noted in 25 (83.3%), neuropsychiatric/cognitive disorders in 26 (86.7.%), palatal abnormalities (including but not limited to palatal cleft) in 18 (60.0%), skeletal abnormalities in 15 (50.0%), allergy in 3 (10.0%), isolated hypoparathyroidism in 11 (36.7%), ophthalmologic abnormalities in 9 (30.0%), neurologic manifestations in 6 (20.0%), dental abnormalities in 8 (26.7%), renal involvement in 5 (16.6%), and hypoplastic thymus in 6 (20.0%).

Associated autoimmune conditions, excluding arthritis, were observed in 8 (26.7%) patients, including uveitis, psoriasis, immune thrombocytopenia (ITP), Evans syndrome, each occurring in one patient. Celiac disease and Hashimoto thyroiditis was each identified in two patients.

Immunological assessment, conducted after the diagnosis of 22q11DS, showed that T-cell counts were decreased in 33.3% of cases (10/30), though all values were above the threshold for complete 22q11DS. B-cell counts were within normal range in all tested children. IgA levels were below the age-adjusted normal range in 13.3% of patients (4/30). T-cell proliferative responses to mitogens were normal in all tested patients, and post-vaccination antibody levels against diphtheria, tetanus, or pneumococcus were within the normal range in all tested individuals.

The median age at arthritis diagnosis was 3 years (range, 1–12 years); 15/30 (50.0%) patients had 4 or fewer affected joints at onset, with a median of 4–5 joints involved initially (range 1–25). The knee was the most frequently involved joint (26 out of 30 patients, 86.7%), followed by the ankle (18/30, 60.0%). Dactylitis of the fingers or toes was observed in 4 out of 30 patients (13.3%). Only one patient developed uveitis. Key characteristics of these patients are detailed in Table 1.

**Table 1:** Demographic and clinical features, treatments, infectious complications, and articular outcomes in 30 patients with 22q11.2 deletion syndrome and arthritis

Pt	Gender	Age at arthritis onset (y)	Family history for arthritis	*N*° of affected joints at onset	Type of affected joints at onset	Uveitis	Laboratory tests					Treatment					Infections before immunosuppressive treatment		Infections after immunosuppressive treatment		Age at last follow-up (y)	Arthritis outcome at last follow up	Erosion on imaging
							↑ ESR	↑ CRP	ANA	CCP	RF	NSAIDs	Oral CS	IACI	DMARDs	Biologics	Mild	Severe	Mild	Severe			
1	M	2	no	1	ankle	no	yes	yes	pos	n.a.	n.a.	yes	yes	yes	no	etanercept	no	yes	no	n.a.	3	Partial remission on medication	no
2	M	3	no	8	elbows, hips, knees, and ankles	no	yes	yes	neg	neg	neg	yes	yes	yes	yes	adalimumab	yes	yes	no	n.a.	23	Active disease	yes
3	F	2	no	7	knees, wrists, ankle, finger IP, toe dactylitis	no	yes	yes	pos	n.a.	neg	n.a.	yes	yes	yes	etanercept, rituximab, abatacept	yes	yes	no	n.a.	16	Active disease	yes
4	F	9	no	18	finger pPeripheral IPs and MCPs, wrist, ankle, TMJ	no	yes	yes	pos	neg	neg	n.a.	yes	n.a.	yes	-	no	no	no	n.a.	11	Remission on medication	no
5	F	7	no	5	knees, ankle, finger dactylitis	no	yes	yes	neg	neg	neg	n.a.	yes	yes	yes	etanercept	no	no	no	n.a.	9	Partial remission on medication	no
6	M	11	no	4	hips, knees	no	yes	yes	neg	neg	neg	n.a.	yes	yes	yes	-	yes	yes	no	yes	18	Active disease	no
7	F	1	no	4	hips, knees	no	yes	yes	pos	neg	neg	yes	no	yes	n.a.	etanercept	no	no	no	n.a.	11	Remission on medication	no
8	F	7	no	4	hips, knees	no	no	no	pos	neg	neg	yes	no	n.a.	yes	abatacept	no	no	no	n.a.	7	Active disease	yes
9	M	5	no	19	wrists, elbows, shoulder, finger IPs and MCPs, knees, ankles, cervical spine	no	no	yes	pos	neg	neg	yes	yes	yes	yes	etanercept, adalimumab, abatacept	no	no	no	n.a.	16	Remission on medication	n.a.
10	F	2	n.a.	7	knees, ankles, cervical spine, finger Peripheral IPs	no	n.a.	n.a.	pos	n.a.	neg	yes	no	n.a.	yes	-	no	no	no	n.a.	16	Remission	no
11	F	3	yes	25	finger IPs and MCPs, wrist, knee, ankles	no	no	yes	pos	neg	neg	n.a.	yes	n.a.	yes	etanercept	no	yes	no	n.a.	29	Active disease	yes
12	F	3	no	19	wrists, elbows, knees, finger MCPs and Peripheral IPs	no	yes	yes	pos	neg	neg	yes	yes	yes	yes	etanercept	no	no	no	n.a.	7	Remisssion on medication	n.a.
13	F	3.5	no	3	knee, wrists	no	yes	n.a.	n.a.	n.a.	n.a.	yes	no	n.a.	n.a.	-	no	no	no	n.a.	15	Remission	n.a.
14	M	12	no	2	ankle, knee	no	yes	yes	pos	n.a.	neg	n.a.	no	n.a.	yes	etanercept	yes	no	no	n.a.	12	Remission on medication	n.a.
15	M	1.5	no	10	elbow, wrists, finger and toe dactilytis, knees, ankles	no	yes	yes	neg	n.a.	neg	yes	no	n.a.	yes	-	no	no	no	n.a.	2	Active disease	yes
16	F	4	no	2	knees	no	no	n.a.	pos	neg	neg	n.a.	no	yes	n.a.	-	no	no	no	n.a.	13	Active disease	yes
17	F	9	yes	1	knee	no	no	no	pos	neg	neg	n.a.	no	yes	n.a.	etanercept	yes	no	no	n.a.	20	Remission on medication	yes
18	F	6	no	6	wrists, knees, ankles	n.a.	yes	n.a.	n.a.	n.a.	n.a.	yes	no	n.a.	n.a.	-	yes	no	no	n.a.	6	Active disease	n.a.
19	F	1.5	yes	5	ankle, knee, wrist, finger Peripheral IPs	no	yes	yes	pos	neg	neg	yes	no	yes	yes	etanercept	no	no	no	n.a.	4	Remission on medication	n.a.
20	M	2	n.a.	4	knees, ankles, cervical spine, fingers Peripheral IPs	no	yes	n.a.	pos	n.a.	neg	yes	no	n.a.	n.a.	-	no	no	no	n.a.	3	Active disease	n.a.
21	M	1	no	17	knees, elbow, wrists, finger MCPs and Perpheral IPs, TMJs	no	n.a.	n.a.	pos	n.a.	neg	yes	yes	yes	yes	-	yes	no	no	n.a.	25	Partial Remission on medication	yes
22	F	2	no	2	knee, ankle	no	n.a.	yes	pos	neg	neg	yes	no	no	no	no	yes	no	yes	no	5	Remission on medication	n.a.
23	F	2	no	8	ankles, shoulder, elbows, wrists	no	n.a.	no	neg	neg	neg	yes	no	no	no	no	yes	no	yes	yes	12	Remisssion on medication	no
24	F	2	no	3	Knees, ankle	no	n.a.	yes	neg	n.a.	neg	yes	yes	yes	yes	etanercept	yes	no	yes	no	16	Active disease	yes
25	F	1.3	no	8	wrist, shoulder, knee, TMJs, finger and toe Peripheral IPs	no	yes	yes	neg	n.a.	neg	yes	yes	yes	yes	etanercept	yes	no	yes	no	14	Remission on medication	no
26	F	12	no	2	knees	no	no	no	neg	n.a.	neg	yes	no	yes	yes	no	no	no	no	no	18	Remission	no
27	F	11	no	4	knee, hip, SI, toe dactylitis	no	no	yes	neg	n.a.	neg	yes	yes	yes	yes	no	no	yes	no	no	14	Active disease	no
28	M	2.3	no	6	knee, elbow, ankles, shoulders	no	yes	yes	pos	n.a.	neg	yes	yes	yes	yes	etanercept, adalimumab	no	no	no	no	13	Remision on medication	no
29	M	2	no	1	ankle	no	yes	yes	pos	n.a.	n.a.	yes	yes	yes	no	etanercept	no	yes	no	no	3	Partial Remission on medication	yes
30	F	3	no	1	knee	yes	yes	yes	pos	n.a.	n.a.	yes	yes	yes	yes	adalimumab	yes	no	yes	no	7	Active disease	no

Abbreviations: ANA – antinuclear antibodies; CCP – cyclic citrullinated peptide; CRP – C-reactive protein; CS – corticosteroid; DMARDs – disease modifying anti-rheumatic drugs; ERS – erythrocyte sedimentation rate; F – female; FU – follow up; IACI – intra-articular corticosteroid injections; IP – interphalangeal joints; M – male; MCP – metacarpophalangeal joints; m – months; n.a – not available; neg – negative; NSAIDs – non-steroidal anti-inflammatory drugs; pos – positive; RF – rheumatoid factor; TMJ – temporo-mandibular joint; y – years

At the onset of arthritis, elevated erythrocyte sedimentation rate (ESR) was documented in most cases (in 18/25, 72.0%, patients in which was available), as were elevated C-reactive protein (CRP) levels (in 20/26, 76.9%, patients in which was available). Antinuclear antibodies (ANA) were present at titers ≥ 1:80 in 19/30 (63.3%) cases. Data available for rheumatoid factor (RF) and cyclic citrullinated peptide (anti-CCP) antibodies showed negative results in all patients tested (25/25 and 14/14 respectively).

Synovial fluid analysis was performed in 7 patients, showing a mild to moderate increase of white blood cells, ranging from 5 to 21.000 × 10^9^/L. Synovial biopsy was performed in two patients, revealing a moderate diffuse lymphocyte infiltration in one case and a chronic diffuse and uniform hyperplastic synovitis with a lymphoplasmacytic infiltrate in the other.

As shown in Table 1, pharmacological treatments included intra-articular joint injections in 14/30 patients (46.7%) with multiple injections needed in 10 of them. Systemic corticosteroids were administered to 16 out of 30 patients (53.3%). Disease-Modifying Antirheumatic Drugs (DMARDS) were used in 20/30 (66.7%), and biologic treatments were used in 17 out of 30 patients (56.7%) – including Etanercept in 14 cases, Adalimumab in four, Abatacept in three, Rituximab in one.

Infections were usually mild and confined to the respiratory tract. Of note, after the initiation of immunosuppressive therapy, infection frequency did not increase for individual patients and was limited to 5 mild upper respiratory infections (two of these patients were on MTX and Etanercept), and two severe infections (one of these patients was on MTX alone). Of these, one patient developed pneumonia requiring intravenous antibiotics in hospital setting. Another patient developed sepsis due to *Pseudomonas aeruginosa*; after three years he was admitted for severe Evans syndrome and macrophage activation syndrome (MAS) requiring intensive immunosuppression complicated by disseminated aspergillosis, hemorrhage, and renal failure from which he subsequently died. Additionally, one patient developed Hodgkin lymphoma at age 25.

Median age at last follow-up was 12.5 years (range 2–29). At last follow-up visit arthritis was still active in 16 out of 30 patients (53.3%), and in remission in the remaing 14 (46.7%), with 11 of those in remission still receiving medication. Among patients with persistent arthritis, eight out of 16 (50%) had at least 3 active joints. Articular damage was present in 12 out of 30 patients (40.0%), with radiologically documented erosions in 10 children. In recent years, magnetic resonance imaging and ultrasound were utilized for assessment, whereas conventional X-rays were primarily used for the first patients included in the study.

## Discussion

Data from the literature show that up to 30% of patients with 22q11DS develop an autoimmune condition, with a prevalence roughly ten times higher than in the general population [8]. Similarly, the reported prevalence of chronic arthritis in 22q11DS is significantly increased compared to the general population [16, 21]. Moreover, as 22q11DS is frequently undiagnosed, the true association with chronic arthritis may be even more underestimated [22].

In our cohort of 30 patients with 22q11DS and chronic arthritis, a female predominance was observed, along with an early onset of joint involvement, occurring mainly before the age of five. A polyarticular distribution was found in 50% of cases, with knees and ankles being the most commonly affected joints.

Arthritis was often accompanied by an increase in inflammatory markers. ANA positivity was frequent but often low-titer, RF and CCP antibodies were consistently negative.

Management was frequently aggressive: many patients required systemic corticosteroids, and most needed DMARD therapy - often methotrexate, typically in combination - highlighting the substantial treatment burden in this cohort. Despite the high proportion of patients treated with MTX and/or biologics, the incidence of infections was not elevated and was generally mild, confined to respiratory tract infections.

The course of arthritis was chronic and severe. Retrospective analysis over a long-term follow-up period, exceeding 10 years in most cases, showed that the disease persisted active in 40% of patients, with two additional cases in partial remission at the last follow-up. Among these, the average number of active joints was four. Patients whose disease onset occurred before 2000, when the use of biological treatments in rheumatology was still limited or unavailable, more frequently experienced persistently active and aggressive disease, with a mean of 8.4 affected joints compared to 5.8 in those with later onset. These findings suggest that early introduction of biologic agents may improve disease control in affected patients, although a complete remission was achieved in about 50% of treated cases. Even among children in remission at the last follow-up, maintenance therapy was commonly necessary. In addition, a considerable percentage already showed radiological joint damage, highlighting the structural impairment despite clinical control.

These findings are consistent with the limited available literature. To our knowledge, 37 pediatric cases of chronic arthritis associated with 22q11DS have been published (Table 2) [12, 14, 16, 19–31]. Nineteen of these cases were reported between 1996 and 2005. Most reports consist of single case studies or small series, while six larger cohorts included at least 30 patients, although most provided limited information specifically on arthritis. The available literature provides limited data on pharmacological management, as most studies were conducted before 2006, when biologic agents were not yet widely available.

**Table 2:** Patients with 22q11.2 deletion syndrome and chronic arthritis reported in the literature

Previous publications: first author, year of publication, case number, reference number	Patients with 22q deletion syndrome and arthritis								
	Gender	Age at arthritis onset (y)	Extended oligo/poly articular arthritis	ANA	RF	ERS/CRP	Uveitis	Treatment	Articular complications
Rasmussen, 1996, 2 patients, with 22qDS and arthritis, [19]	F	7 yrs	yes	positive	positive	increased	negative	unknown	yes
	F	5 yrs	yes	positive	negative	increased	negative	NSAIDs	yes
Sullivan, 1997, 80 patients with 22qDS of which 3 with arthritis [16]	F	5 yrs	yes	negative	negative	unknown	unknown	unknown	unknown
	M	19 m	yes	positive	negative	increased	unknown	NSAIDs	unknown
	M	17 m	yes	positive	negative	increased	unknown	NSAIDs	unknown
Di Rocco, 1998 1 patient with 22qDS and arthritis, [23]	F	3.5 yrs	yes	unknown	unknown	unknown	unknown	NSAIDs, corticosteroids, MTX	unknown
Verloes, 1998 3 patients with 22qDS and arthritis [22]	F	4 yrs	yes	unknown	unknown	increased	negative	NSAIDs, corticosteroids	yes
	M	3 yrs	yes	negative	negative	unknown	unknown	gold, Plaquenil, MTX	yes
	F	1 yrs	yes	unknown	unknown	increased	unknown	salicylates	yes
Jawad, 2001 195 patients with 22qDS of which 1 with arthritis [12]	F	unknown	No (oligoarticular)	unknown	unknown	unknown	unknown	unknown	unknown
Pelkonen, 2002 3 patients with 22qDS and arthritis [21]	M	8 m	yes	negative	negative	unknown	negative	unknown	unknown
	F	12 m	yes	negative	negative	unknown	negative	unknown	unknown
	F	17 m	yes	negative	negative	unknown	negative	unknown	unknown
Gennery, 2002 32 patients with 22qDS of which 1 with arthritis [24]	F	9 yrs	No (oligoarticular)	unknown	negative	unknown	unknown	unknown	unknown
Davies, 2003 5 patients with 22qDS and arthritis [14]	F	6 yrs	yes	negative	positive	increased		intra-articular/systemic corticosteroids, MTX	yes
	F	11 yrs	yes	positive	negative	negative	negative	NSAIDs, MTX, hydroxychloroquine	yes
				low titer					
	M	3 yrs	yes	positive	negative	negative	negative	systemic corticosteroids, MTX	no
				low titer					
	F	1.5 yrs	yes	positive	unknown	negative	negative	systemic corticosteroids, MTX, surgical interventions	yes
				low titer					
	F	5 yrs	yes	negative	negative	negative	negative	NSAIDs, MTX	yes
Sato, 2011 1 patient with 22qDS and arthritis [25]	F	4 yrs	yes	negative	negative	increased	unknown	NSAIDs, MTX	unknown
Tison, 2011 130 patients with 22qDS of which 2 with arthritis [26]	F	4 yrs	No (oligoarticular)	positive	unknown	unknown	unknown	unknown	unknown
	F	3 yrs	yes	unknown	unknown	unknown	unknown	unknown	unknown
Cancrini, 2014 228 patients with 22qDS of which 5 with arthritis [27]	5 cases, not further described								
Salehzadeh, 2014, 1 patient with 22qDS and arthritis [28]	M	unknown	No (oligoarticular)	negative	negative	increased	negative	unknown	unknown
Giardino, 2019 283 patients with 22qDS of which 6 with arthritis [29]	6 cases, not further described								
Sestan, 2020 1 patient with 22qDS and arthritis [20]	M	unknown	yes	negative	negative	increased	negative	NSAIDs, MTX, corticosteroids, IVIGs, TNFi	yes
Belina Y. Yi, 2023 1 patient with 22qDS and psoriatic arthritis [30]	F	15y	yes	unknown	unknown	unknown	no	ustekinumab	no
de Oliveira-Sobrinho, 2024 1 patient with 22qDS and psoriatic arthritis [31]	M	3y	yes	negative	negative	negative	no	unknown	yes

Abbreviations: ANA – antinuclear antibodies; CRP – C-reactive protein; ERS – erythrocyte sedimentation rate; F – female; IVIG – intravenous immunoglobulin; M – male; m – months; MTX – methotrexate; NSAIDs – non-steroidal anti-inflammatory drugs; RF – rheumatoid factor; TNFi – tumor necrosis factor inhibitor; yrs – years.

There is ongoing debate about whether the chronic arthritis observed in patients with 22q11DS should be considered a form of juvenile idiopathic arthritis (JIA), the most common type of chronic arthritis before the age of 16.

Based on data from larger cohorts of patients with 22q11DS, chronic arthritis has been reported in 1.5% to 3.7% of cases, significantly higher than the estimated prevalence of JIA in the general population, which ranges from 0.0038% to 0.4% [26, 27].

Furthermore, chronic arthritis in 22q11DS presents a distinctive clinical profile, predominantly characterized by a polyarticular pattern and elevated inflammatory markers. ANA testing is not uncommonly negative or present at low titers, and RF and CCP are consistently negative. Synovial biopsy, performed in a few cases, revealed chronic or acute non-specific inflammation [16]. Disease onset typically occurs before the age of five, with as many as 20% of patients in the cohort described by Sullivan et al. presenting before the age of one year [16]. The disease course is often severe, requiring multiple treatments and frequently resulting in joint damage.

Additionally, although uveitis is the most common extra-articular manifestation of JIA, typically presenting as a chronic anterior non-granulomatous inflammation affecting the iris and ciliary body of both eyes [32], no cases have been reported in the literature to date in pediatric patients with 22q11DS and chronic arthritis. Only one case of isolated idiopathic bilateral chronic granulomatous panuveitis has been described in a 25-year-old man with 22q11DS [33]. Therefore, the only reported instance of chronic arthritis in 22q11DS associated with bilateral uveitis, further complicated by synechiae and cataract, is the one in our cohort.

In addition to arthritis, autoimmunity in 22q11DS may present with a wide range of manifestations.

In our cohort, autoimmune conditions other than arthritis were observed in approximately 27% of patients. These included immune thrombocytopenic purpura (ITP), Evans syndrome, uveitis, psoriasis, Hashimoto’s thyroiditis, and celiac disease. Similar findings have been reported in the literature [12, 34–44], highlighting the broader spectrum of immune dysregulation associated with 22q11DS. Although recent studies have proposed a potential autoimmune basis also for the neuropsychiatric and cognitive features of 22q11DS [13], no clinical, radiological, or biochemical evidence supporting this hypothesis was identified in our cohort, despite such symptoms being present in most patients.

The mechanisms underlying autoimmunity in 22q11DS remain poorly understood [43], and several immunological abnormalities can be implicated. Severe T-cell defects -including reduced naive CD4 + T cells, low cytotoxic CD8 + T cells, and an inverted naive/memory T-cell ratio - may compromise both central and peripheral tolerance [12, 44–49]. In particular, reduced thymic epithelial cell output and possibly decreased expression of the autoimmune regulator (AIRE) in medullary thymic epithelial cells may impair negative selection, allowing autoreactive clones to escape into the periphery [3, 8, 10]. The limited TCRs observed in many patients further restricts the extent of self-tolerance, while Tregs are often deficient in terms of quantity and function, weakening peripheral control of autoreactive responses. Furthermore, chronic T cell lymphopenia enhances homeostatic proliferation and repertoire imbalance [9, 11, 44, 48, 49]. Notably, Sullivan et al. reported that patients with arthritis were among the most immunocompromised in their cohort [16]. IgA deficiency, which is frequently observed in children with 22q11DS and arthritis, may reflect underlying T-cell dysregulation and has also been associated with a broader predisposition to autoimmunity [24–25; 50]. Furthermore, an alteration in B cell homeostasis has often been documented, with a reduction in switched memory B cells and elevated levels of B cell activating factor, which create a survival niche for autoreactive B cells and promote autoantibody production. A context of chronic low-grade immune activation, sustained by persistent lymphopenia and impaired immune regulation, may act synergistically to promote immune dysregulation [3, 9, 11, 24, 29, 48, 49, 51].

Although no definitive genetic predisposition has been identified, it has been hypothesized that polymorphisms on the intact chromosome may unmask susceptibility loci in the hemizygous state [2]. Moreover, the deleted 22q11.2 region commonly includes the TBX1 gene, a key regulator of progenitor cell differentiation and immune development [3, 52].

This study has several limitations. First, its retrospective design and reliance on clinical records may have introduced variability in data completeness and assessment methods across centers. Second, the absence of longitudinal immunological evaluations limited our ability to explore correlations between immune dysfunction and disease course. Third, the relatively small sample size and the likely referral bias toward more severe or complex cases reduce the generalizability of our findings. Additionally, the lack of a control group, either patients with 22q11DS without arthritis or patients with JIA without 22q11DS, limits the ability to define the specific characteristics of arthritis in this genetic context. The therapeutic approaches were not standardized, further complicating interpretation of treatment responses. Moreover, we aknowledge the lack of consistent data on ANA characterization with specific assays (such as anti-dsDNA and anti-ENA); however, based on the long-term clinical follow-up, no signs or symptoms suggestive of other systemic autoimmune diseases have been observed. Finally, imaging and histological data were available only for a minority of patients.

Nevertheless, this relatively large international cohort provides important clinical evidence suggesting that chronic arthritis in patients with 22q11DS may represent a distinct inflammatory phenotype. Future prospective studies involving larger cohorts, standardized immunological monitoring, and appropriate comparison groups are warranted to better characterize the pathogenesis, guide therapeutic strategies, and evaluate long-term outcomes.

## Conclusions

Chronic arthritis in patients with 22q11DS is a relatively frequent, yet likely under-recognized complication. It appears to stem from the complex immunological dysregulation associated with the syndrome, although its precise pathogenic mechanisms remain unclear.

Arthritis in this context is characterized by early onset, polyarticular involvement, rare association with uveitis, elevated inflammatory markers. The clinical course is prolonged, poorly responsive to conventional therapies, and may lead to joint damage and functional impairment.

Further studies are warranted to better define optimal management strategies and clarify the long-term prognosis.

## Data Availability

The datasets used and/or analysed during the current study are available from the corresponding author on reasonable request.

## Abbreviations

22q11DS: 22q11 Deletion Syndrome
ANA: Antinuclear Antibodies
anti: CCP–Anti–Cyclic Citrullinated Peptide Antibodies
CRP: C–Reactive Protein
DMARDs: Disease–Modifying Antirheumatic Drugs
ESR: Erythrocyte Sedimentation Rate
FISH: Fluorescence In Situ Hybridization
IEI: Inborn Errors of Immunity
IgA: Immunoglobulin A
IgE: Immunoglobulin E
IgG: Immunoglobulin G
ITP: Immune Thrombocytopenic Purpura
JIA: Juvenile Idiopathic Arthritis
MAS: Macrophage Activation Syndrome
MLPA: Multiplex Ligation–dependent Probe Amplification
MTX: Methotrexate
RF: Rheumatoid Factor
TCR: T Cell Receptor
TREC: T–cell Receptor Excision Circles
Treg: Regulatory T Cell
